# Entropy Estimation Using a Linguistic Zipf–Mandelbrot–Li Model for Natural Sequences

**DOI:** 10.3390/e23091100

**Published:** 2021-08-24

**Authors:** Andrew D. Back, Janet Wiles

**Affiliations:** School of Information Technology and Electrical Engineering, The University of Queensland, Brisbane, QLD 4072, Australia; j.wiles@uq.edu.au

**Keywords:** entropy estimation, Zipf–Mandelbrot–Li law, language models, probabilistic natural sequences

## Abstract

Entropy estimation faces numerous challenges when applied to various real-world problems. Our interest is in divergence and entropy estimation algorithms which are capable of rapid estimation for natural sequence data such as human and synthetic languages. This typically requires a large amount of data; however, we propose a new approach which is based on a new rank-based analytic Zipf–Mandelbrot–Li probabilistic model. Unlike previous approaches, which do not consider the nature of the probability distribution in relation to language; here, we introduce a novel analytic Zipfian model which includes linguistic constraints. This provides more accurate distributions for natural sequences such as natural or synthetic emergent languages. Results are given which indicates the performance of the proposed ZML model. We derive an entropy estimation method which incorporates the linguistic constraint-based Zipf–Mandelbrot–Li into a new non-equiprobable coincidence counting algorithm which is shown to be effective for tasks such as entropy rate estimation with limited data.

## 1. Introduction

Natural systems such as language, can be understood in terms of symbolic sequences described within an information-theoretic framework, where meaning is encoded through the arrangement of probabilistic elements. When placed in a mathematical framework, we can characterize and begin to understand the meaning of messages, not only on the basis of the meaning directly attached to words, but on the statistical characteristics of symbols.

Using this approach, natural language can be viewed as observing one or more discrete random variables *X* of a sequence *X* = $X_{1},\ldots ,X_{i},\ldots ,X_{K}$ $,X_{i}=x\in X^{M},$ that is, $x_{i}$ may take on one of *M* distinct values, $X^{M}$ is a set from which the members of the sequence are drawn, and hence $x_{i}$ is in this sense symbolic, where each value occurs with the probability $p\left(x_{i}\right),i\in \left[1,M\right].$

The single symbol Shannon entropy (if not otherwise specified, any reference to entropy will refer to the classical Shannon entropy of unigram probabilities.) is defined for unigrams as [1,2]

(1) $$H_{0}\left(X\right)=-\sum_{i=1}^{M}p\left(x_{i}\right)\log_{2}\left(p\left(x_{i}\right)\right)$$

In the context of language processing, statistical models of symbol sequences are of interest and can be defined by the probability $p\left(\Omega \right)=p\left(s_{1},\ldots ,s_{N}\right)$ where Ω is a sequence of *N* symbols $\left\{s_{i}\right\}.$ If the full sequence is available, the n-gram entropy can be directly computed using the same formula as (1), where instead of computing unigram probabilities, the joint probabilities are estimated so the n-gram entropy is obtained. However, the problem with this approach is that a large amount of data is generally required and the reliability is questionable for N>5 [3].

In probabilistic language modeling, it is often of interest to predict the next symbol in a sequence using the previous symbols. Hence, the joint probability can be computed using the Markov property by considering the previous block of symbols in terms of the conditional probabilities as

(2) $$p\left(s\right)=\prod_{i=1\ldots N}p\left(s_{i}|s_{1}\ldots ,s_{i-1}\right)$$

Hence, provided the symbol probabilities can be estimated, it is possible to determine the n-gram probabilities and n-gram entropy of language sequences.

A related method of characterizing language models is to measure perplexity [4], defined as

(3) $$P_{e}\left(s\right)=2^{H(s)}$$

In contrast to entropy, which can be understood as measuring the average number of bits to encode the information in a symbol, perplexity can be intuitively understood as measuring the total number of bits required to encode the information in a sequence; hence, the smaller the value the better.

While entropy provides a measure of information in a given sequence, this raises the question of how quickly the information grows with increasing text length [1,5]. The idea is that the entropy rate can measure the complexity of language by the average information content of symbols such as words taken over a sufficiently long period. Similarly, the effectiveness of compression algorithms can be measured by how closely the algorithm can compress any stationary and ergodic source down to the entropy rate for a sufficiently long input source sequence [6].

Entropy rate has been of interest for analyzing the information content neuronal spike sequences [7], complexity of short heart period variability [8], attention models using visual salience attention [9], complexity of animal vocal complexity [10], statistical structure of non-redundant coding sequences in DNA [11], behavior prediction [12] and in estimating the long-term memory of language models [13].

A problem with entropy estimation is characterizing infrequently occurring symbols, hence requiring a potentially large number of samples to adequately model the probabilistic structure [14].

In contrast to statistical descriptive applications which depend on large amounts of data, we are interested in building models of social interaction using entropy estimation methods with limited available data.

Various efficient methods of entropy estimation have been proposed. Entropy estimation over short symbolic sequences for dynamical time series models was considered in [15]. A computationally efficient method for calculating entropy based on a James–Stein-type shrinkage estimator was proposed in [16]. Methods for overcoming bias in maximum likelihood entropy estimators with limited data have been examined in [17]. The Nemenman–Shafee–Bialek (NSB) entropy estimator extends this concept to correct sample size-dependent bias by using a Bayesian approach to construct priors with power–law dependence on the probabilities, in particular, using Dirichlet distributions [18].

The advantages of more sophisticated entropy estimation techniques which go beyond naive plug-in methods are evident. These can be broadly referred to as “model-based” because they introduce some additional complexities into an otherwise simple algorithm which takes into account some understanding of the nature of the data and the estimation process whilst remaining broadly applicable. For example, a model-based estimator using an understanding of how sequences of symbols will have probabilistic patterns of “coincidence” was proposed in [19].

In this paper, we consider a novel probabilistic model-based entropy estimator which extends [19] and is comparable to [18] in that it uses a limited amount of data and an a priori model as a basis for constructing an efficient entropy estimator. The model “hint” that we introduce is the idea that for many natural sequences including language, instead of a naive estimator, the probabilistic distribution of symbols is expected to follow linguistic patterns. Hence, the basis for our proposed approach is to develop an analytic rank-based Zipfian-style probabilistic model which is constrained to accommodate the linguistic features of human language and to incorporate this into an efficient non-equiprobable coincidence counting the entropy estimation algorithm.

In the next section, we describe a coincidence-counting entropy estimator and introduced the concept of linguistic entropy. In Section 3, we introduce a new framework of linguistic probabilistic models which is incorporated into the proposed entropy estimator. In Section 4, we demonstrate the efficacy of the proposed model and show that it provides a high degree of accuracy while requiring a small number of samples.

## 2. Model-Based Entropy Estimation

### 2.1. Coincidence Counting Approach

Entropy estimation difficulties can occur with low probability events exacerbated due to real-world issues surrounding the data, including problems of small data sets [20], limited resource environments [21], bias due to heavy-tailed distributions [22] and uneven distributions with poorly populated bins [23]. The latter problem is especially evident in estimating entropy in language involving very low probability events such as infrequent words.

The problem of undersampling in the context of entropy estimation, where the alphabet size is large compared with the number of samples, that is, N≪M, has been considered at length where it is well known that significant bias can occur, particularly in the case of using binning approaches [23,24]. Hence, alternative entropy estimation algorithms are of interest which can provide useful results with a small number of samples [25,26,27].

One class of proposed solutions is based on the method of coincidence counting to derive entropy from the phase space trajectory of symbolic events [28]. In particular, Ma proposed the method of coincidence counting as a suitable method of deriving entropy from the phase space trajectory, noting the problematic issue of metastability with estimating the empirical probability distribution. A simple algorithm for entropy estimation based on this approach was proposed in [19]. Their novel approach used the idea of estimating probabilities from a quadratic function of the inverse number of symbol coincidences; however, it has the limitation of this method, which was that it assumed equiprobable symbols. In the next section, we show how it is possible to extend this to the non-equiprobable case by using an analytic Zipf–Mandelbrot–Li law.

### 2.2. Linguistic Entropy Estimation

Consider a sequence of symbols which is defined by a discrete (or symbolic) random variable *x* which may take on a finite number *M* of distinct values $x_{i}\in \left\{x_{1},\ldots ,x_{M}\right\}$ with probabilities $p\left(x_{i}\right),i\in \left[1,M\right].$ For example, suppose M=4 and we have a sequence of symbols abcdabc. The frequency of symbols can be estimated as a function of the distance between consecutive repeating symbols or the ‘coincidence distance’ and in this case, the initial distance for *a* is D(a;M)=5. Hence, it can be observed that by measuring this distance, it may be possible to estimate the relative frequency of any given symbol by measuring the distance between them.

To compute the probability f(n;M) of a first coincidence occurring exactly at the *n*th symbol for 1<n<M means that it is necessary to compute the probability of drawing no repeating symbols in the entire sequence up to the (n−1)th draw given by $\tilde{F}\left(n-1;M\right)$ and consequently drawing any $q_{n-1}\in \left[2,\ldots ,n-1\right]$ identical symbols is given by F(n−1;M). Hence, the *n*th symbol coincidence probability is given by [19]

(4) f(n;M)=F(n;M)−F(n−1;M)

The expectation of the discrete parameter *n* and its associated probability f(n;M) is given by

(5) $$\begin{array}{cc} E[n] & =J(n;M) \end{array}$$

(6) $$\begin{array}{cc} \; & =\sum_{n=0}^{M}nf\left(n;M\right) \end{array}$$

Since *n* is a function of M, we may define the coincidence distance:

(7) D(M)=E[n].

By forming a model to estimate *M* using the symbol distance *D* such as

(8) $$\begin{array}{cc} \hat{M}\left(D\right) & =G\left(\Theta ;D\right) \end{array}$$

(9) $$\begin{array}{cc} \; & =\sum_{i=0}^{n_{p}}\theta_{i}D^{i} \end{array}$$

then by measuring D(M) and hence evaluating *M* from the parametric model, then the entropy can be directly estimated from the symbol coincidences.

The model parameters $\theta_{i}$ can be determined by fitting a curve to an ensemble of data. For equiprobable symbols, the Shannon entropy is estimated as

(10) $$H_{0}\left(M\right)=\log_{2}\left(\hat{M}\left(D\right)\right)$$

Using this approach with only a small number of symbol observations, entropy estimation for equiprobable symbols was shown to be accurate and with a low bias of [19,29].

Now, for any given M, each symbol of a specified rank *r* can be treated as being equiprobable and hence by considering the probability of each ranked symbol, then we have:

(11) $$\tilde{F}\left(n;M\right)=1\cdot \left(1-P_{2}\right)\cdot \left(1-P_{3}\right)\cdots \left(1-P_{n-1}\right)$$

where $\tilde{F}\left(n;M\right)$ is the probability of drawing any symbol on the first try followed by any other different symbol up to the *n*th draw and up to *n* symbols, and $P_{h}$ is the probability of independently drawing h−1 identical symbols from a set of *M* in h−1 draws.

It follows that we can define the probabilities in terms of rank using a probabilistic model such as the Zipf–Mandelbrot–Li law developed by Li [30], who showed that the constants can be computed as

(12) $$\begin{array}{cc} \alpha & =\frac{\log_{2}\left(M+1\right)}{\log_{2}\left(M\right)} \end{array}$$

(13) $$\begin{array}{cc} \beta & =\frac{M}{M+1} \end{array}$$

(14) $$\begin{array}{cc} \gamma & =\frac{M^{\alpha -1}}{{\left(M-1\right)}^{\alpha }} \end{array}$$

with a normalization step introduced in [14] as

(15) $$\gamma^{\prime }=\frac{\gamma }{\kappa }$$

to give:

(16) $$\sum_{i=1}^{M}p\left(i\right)=1,\;\sum_{i=1}^{M}\frac{\gamma }{{\left(r+\beta \right)}^{\alpha }}=\kappa$$

which leads to:

(17) $$P\left(r;M\right)=\frac{\tilde{\gamma }}{{\left(r\left(L\right)+\tilde{\beta }\right)}^{\tilde{\alpha }}}$$

**Figure 1:**
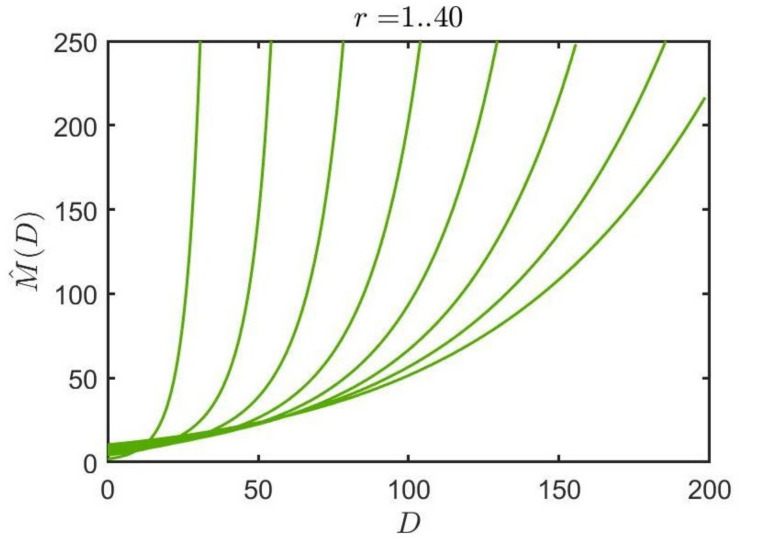
The inverse model curves (1..40) for the proposed algorithm are shown here for the range of the top 40 ranked symbols, also shown here for an alphabet size of M=200.

This approach provides an equiprobable representation of the symbols by considering a different model for each symbol rank. Hence, an invertible rank-based model for $D\left(M\right)=J^{\prime }\left(n,r,M\right)$ such as a power-based model is chosen so that the inverse model can be directly estimated using the forward model, for example, as

(18) $$\hat{D}\left(M;r\right)=\frac{1}{a}{\left(\hat{M}\left(r;D\right)-c\right)}^{\frac{1}{b}}$$

where $\theta =\left\{a,b,c\right\}$ are the forward parameters. Given an estimate $\hat{M}\left(D;r\right)$ from the observed inter-symbol distance, it is possible to apply this parameter to the Zipf–Mandelbrot–Li set of equations in addition to our rank-based probability model, and estimate the entire set of symbolic probabilities. Using ${\hat{P}}_{h}\left(r,M\right),$ the entropy can then be easily estimated as

(19) $${\hat{H}}_{1}\left(r,X\right)=-\sum_{h=1}^{\hat{M}}{\hat{P}}_{h}\left(r,M\right)\log_{2}\left({\hat{P}}_{h}\left(r,M\right)\right)$$

which defines the rank *r* Shannon entropy estimate. The model is applied by determining the mean distance between symbols $D_{i}\left(r\right)$ and then finding the estimated value $\hat{M}\left(D;r\right)$ which is used to estimate entropy. The scaling of the inverse model curves for the proposed algorithm can be observed in Figure 1.

### 2.3. Remarks on Bias and Convergence Properties

The proposed algorithm is defined in terms of a Zipf–Mandelbrot–Li distribution which uses a coincidence counting approach to estimate the mean symbolic distance between one or more ranked symbols and then use this to form an estimate of the whole distribution. We can consider the bias and convergence properties in terms of the maximum likelihood estimator for $\hat{D}\left(M;r\right)$ in contrast to the probabilities directly in a plug-in entropy estimator.

Algorithmic bias can occur due to systematically underestimating the mean distance between symbols $D_{i}\left(r\right)$ [29]. To compute the bias precisely requires a closed form of the probability density function of $D_{i}\left(r\right).$ Analyzing this requires considering (4) and (11)–(16), where an approximation to the probability distribution can be obtained from the multiplicative process defined by (11) in terms of a log-normal distribution [31]. However, since a closed form solution for the likelihood of a log-normal distribution is not generally available, it is non-trivial to determine the specific bias properties [32,33]. One possible solution to this is to examine the probabilistic bounds [34]. Though we do not consider it in this paper, the bias properties of the proposed algorithm may also be improved by applying techniques such as the Miller–Madow procedure [16].

In terms of the convergence properties of the algorithm, a full proof of convergence properties is beyond the scope of this paper; however we provide an indication of some properties of the expected result. Note that $D_{i}\left(r\right)$ can be determined from the maximum likelihood estimation of the inter-symbol distance for any given symbol. Choosing the most frequent symbol r=1, then $D_{i}\left(1\right)$ will converge in the sense of a usual maximum likelihood estimator to within some limits with a particular confidence level [35].

Convergence depends on symbols with rank r=1, with specified probability ${\hat{P}}_{h}\left(1,M\right)$ and all other possible symbols with probability $1-{\hat{P}}_{h}\left(1,M\right).$ Hence, the number of symbols required to estimate $D_{i}\left(r\right)$ to within the specified degree can be found by means of the Dvoretzky–Kiefer–Wolfowitz (DKW) inequality [35].

Since the convergence of $D_{i}\left(r\right)$ depends on the estimation of ${\hat{P}}_{h}\left(r,M\right)$, then the DKW inequality provides the following result [14]:

(20) $$P\left\{\underset{r\in N}{\sup}\left|{\hat{P}}_{h}\left(r,M\right)-P\left(r,M\right)\right|<\epsilon_{r}\right\}\leq \zeta^{\prime }$$

where for a maximum difference $\epsilon_{r}$ between the estimated probability ${\hat{P}}_{h}\left(r,M\right)$ and its theoretical target value $P\left(r,M\right),$ there will be $n_{r}$ samples required to estimate the probability with a confidence level of $\zeta^{\prime },$ specified by

(21) $$\zeta^{\prime }=1-2e^{-2n_{r}\epsilon_{r}^{2}}$$

Hence, following [14], it can be shown that for a given confidence level, the minimum number of samples required to estimate $P\left(r,M\right)$ which are described by a Zipf–Mandelbrot–Li approximation, can be found as

(22) $$N_{r}\leq \frac{8}{P\left(r,M\right)\Delta_{r}^{2}}\ln\left(\frac{2}{1-\zeta^{\prime }}\right)$$

where for a ranked distribution, $\Delta_{r}$ is found as

(23) $$\Delta_{r}=P\left(r,M\right)-P\left(r+1,M\right)$$

Now, it follows that the relative convergence performance can be defined in terms of a scaling factor $\lambda_{f}\left(M\right)$ which measures the reduction in samples required for convergence compared to a naive plug-in estimator as measured against the symbolic alphabet size M. Hence, we have:

(24) $$\lambda_{f}\left(M\right)=\frac{\frac{8}{P\left(M,M\right)\Delta_{M}^{2}}\ln\left(\frac{2}{1-\zeta^{\prime }}\right)}{\frac{8}{P\left(1,M\right)\Delta_{1}^{2}}\ln\left(\frac{2}{1-\zeta^{\prime }}\right)}$$

which simplifies to:

(25) $$\lambda_{f}\left(M\right)=\frac{P\left(1,M\right)\Delta_{1}^{2}}{P\left(M,M\right)\Delta_{M}^{2}}$$

**Figure 2:**
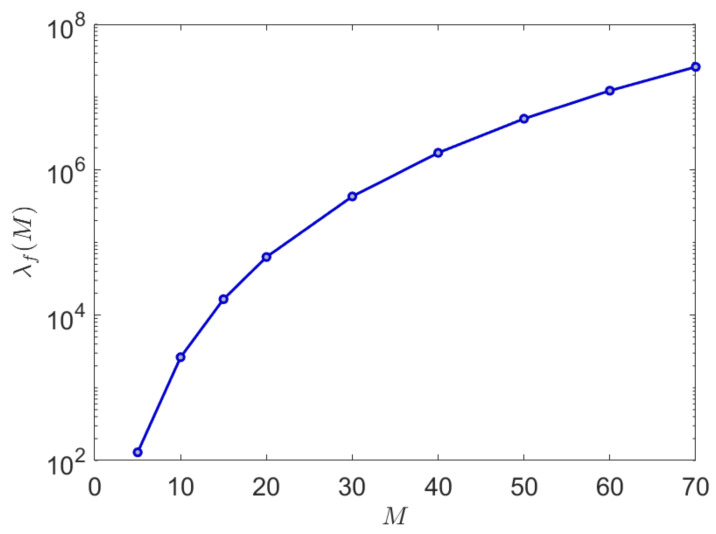
This graph shows the relative convergence performance $\lambda_{f}\left(M\right)$ of the proposed algorithm scaled against a conventional plug-in entropy estimation algorithm as measured against the symbolic alphabet size M. Note that the improvement is easily several orders of magnitude for alphabet sizes of interest in the range 20–40.

A graph of the relative convergence performance is shown in Figure 2 where an improvement of several orders of magnitude in the reduction in the number of samples required can be observed for alphabet sizes in the ranges M=20−40 which are of typical interest in both natural and synthetic languages.

In contrast, the conventional plug-in estimator requires an estimation of all symbol probabilities which depends on the probabilities of all symbols and consequently significantly more samples. A full derivation of this latter result is shown in [14]. Hence, the proposed entropy estimation algorithm converges with a factor of approximately $\lambda_{f}\left(M\right)$ fewer symbols than in the conventional case.

The current estimator employs a ZML distribution, and in the next section, we extend this model to include linguistic constraints.

## 3. Linguistic Probabilistic Models

### 3.1. Limitations of Zipfian Models for Language

For various natural sequences, Zipf’s law describes how the frequency of ranked events occurring in such a way that they can be described by a power law [36,37,38]. This question of whether Zipf’s law is a universal law of natural language and other phenomena has generated substantial interest over a long period of time [39].

The premise of Zipf in 1949 was that natural systems follow a principle of least effort, which means that individuals will follow a course of action which involves the expenditure of the least amount of work [40]. In terms of human language, this implies that the distribution of word use would follow the same principle so communication would occur efficiently with the least effort.

Various ongoing works have attempted to prove and disprove results in this field. Miller proposed that a monkey typing would produce a natural language with Zipfian distribution [41]. This argument was based on the result that the probabilistic distribution of words in natural languages only occurs as a statistical artifact of random spaces and can be described by Zipf’s law.

While this claim has continued to generate considerable interest decades later, in fact, Miller’s result was shown to be flawed by Howes in 1968 [42]. The problem with Miller’s result is that assumes all word probabilities are strictly ranked by word length. Moreover, it assumes that all possible words of the same length have the same probability and that all sequences of letters are equiprobable. Clearly, these assumptions are not valid for natural language. A more recent analysis of this problem was performed, for example, considering unequal letter probabilities and log normal rank distributions [43,44]. It was found in [45] that the average information content was more consistently ranked than word length, by examining the inter-word statistical dependencies as the n-gram entropy of words in a local linguistic context.

Cancho and Sole considered the principle of least effort in human language as a compromise between speaker’s and hearer’s needs, where they were able to show results which indicate how Zipf’s law explains the observations [46]. The concept of efficiency in languages was considered in [47], where efficiency can be defined in terms of successfully transmitting many different messages with minimal effort, yet balanced in terms of informativeness and complexity. Principles of least effort are closely related to the concept of semantic language universals by which such effort can be instantiated and measured. Accordingly, the principle of *ease of learning* was found to have strong evidence as a language universal in [48].

Zipf’s law has been shown to occur as a result of the choice of rank as an independent variable [30,49], and hence has been challenged in terms of suitability as a universal model of human language or other natural sequences [50]. For example, in [30], it was reported that because the word frequency distribution of random texts can exhibit Zipfian characteristics, then Zipf’s law is unsuitable as a criterion for identifying natural languages. However the statistical analysis of this result was disputed in [51] where it was shown that the rank distributions of random and natural texts are statistically inconsistent, and this suggests that Zipf’s law may exist as a fundamental principle in natural languages.

While word frequency has generated significant interest, Zipf’s law may operate at other levels in natural language, for example some results show the ranked order of phrases in natural language [52]. Moving towards more complex understandings of how Zipf’s law can be refined, Corral considered the issue of how Zipf’s law applies to normalized language element lemmas, i.e., a stem-like word form [50], and how a more complex formulation of Zipf’s law of word frequency arising from a mixture of conditional distributions of frequency at different lengths may provide a better explanation of observations [53].

It is evident that there is not yet a single definitive answer as to the question of whether Zipf’s law is necessarily a universal model of human language. However, it is clearly useful in forming a model of ranked symbolic information transmission which mimics human language elements [49,53] and have proved helpful as a probabilistic model for characterizing the observed behavior of natural symbolic sequences [54]. Here, we do not seek to prove the universality of Zipfian laws for language, but we consider their use as a way to model some aspects of natural language and how this may be useful for entropy estimation.

While a number of variations of Zipf’s law have been proposed, including the Bradford Law [55] and Lotka Law [56], we previously proposed a new variation of the model we refer to as the Zipf–Mandelbrot–Li law [14,30,57,58,59,60], which models the frequency rank *r* of a language element $x\in \Sigma^{M+1}$ from an alphabet of size M+1, then, for any random word of length L, given by $v_{k}\left(L\right)=\left\{w_{s},x_{1},\ldots ,x_{L},w_{s}\right\},\;k=1,\ldots ,M^{L}$ the frequency of occurrence is determined as

(26) $$p_{i}\left(L\right)=\frac{\lambda }{{\left(M+1\right)}^{L+2}}\;i=1,\ldots ,M^{L}$$

where Li showed that λ can be analytically determined [30].

While these various rank-based laws provide a convenient analytical framework to model symbolic sequences, there is a problem in terms of known languages because such simple models carry no particular linguistic information. For example, consider the case of five-letter words. In English, there are approximately 12,500 known five-letter dictionary words. However, in the unconstrained ZML model, there can be 11.8 M words allowed. In the unconstrained form, this means that we allow words such as aaaag, rrrrx, czzzs, xyyaa and many other invalid words. A way to understand this is that such words do not conform to known linguistic principles such as orthographics [61], syllable structure [62], consonant/vowel ratio and organization [63,64], letter position [65] and graphemes [66]. Each of these can be viewed as a constraint on the allowable letters and their position in any word and hence it is evident that an unconstrained word-letter model allows a greater number of words than should be anticipated.

This raises the question of whether it is possible to introduce a probabilistic model which extends the advantageous Zipf–Mandelbrot–Li law to one which includes some linguistic constraints. Such a model would potentially provide the same useful analytic formulation while providing a more realistic bound on the types of words implicitly permitted. While the ZML model offers an effective basis for computing linguistic behavioral characteristics, it is evident that there is a need for improved models which provide a greater degree of conformity to the true properties of human language if such models are to be better utilized.

### 3.2. Unconstrained Rank-Ordered Probabilistic Model

Given a natural sequence such as language elements, consider a Zipfian probabilistic model of symbolic events which models the frequency rank *r* of a word (a word or n-gram is not necessarily referring to human language, but indicates a specific set of sequentially occurring symbols.), i.e., the *r*-th most frequent word, by a simple inverse power law, such that the frequency of a word f(r) scales according to an equation which is given by

(27) $$f\left(r\right)\propto \frac{1}{r^{\alpha }}$$

where a proportionality-dependent constant on the particular corpus may be introduced, Ref. [30] and where typically α≈1. Thus, if $p_{i}\left(x\right)$ follows a Zipfian law, then $p_{0}\left(x\right)\propto 1/M$ and $p_{i}\left(x\right)=\varphi f\left(r\right).$ This power law equation can be understood in terms of the principle of least effort. It indicates that frequency decays linearly as the rank increases according to a log–log scale.

A way to view this is that according to Zipf’s law, efficient language will minimize the effort between speaker and hearer [46]. Hence, the speaker may use a small vocabulary of common words to minimize effort in speaking and the hearer may desire a large vocabulary of less common words to minimize the effort in terms of ambiguity or confusion (Note that ambiguity and confusion are different concepts. The former may define the meaning of words, whereas confusion can relate to the intelligibility of words. Zipf’s law provides a mathematical explanation of the balance between these competing features.).

We consider the Zipf–Mandelbrot law below [58]:

Given symbols $x\in \Sigma^{M+1}$ from an alphabet of size M+1 which includes a blank space $w_{s}$ then for any random word of length L, given by $v_{k}\left(L\right)=\left\{w_{s},x_{1},\ldots ,x_{L},w_{s}\right\},$ $k=1,\ldots ,M^{L}$ the total number of words possible is given by

(28) $$\begin{array}{cc} N_{w} & =\prod_{k=1}^{L}M_{k},\;k=1,\ldots ,L\\ \; & =M^{L} \end{array}$$

It follows that the frequency of occurrence for an unconstrained word of length *L* following a Zipf–Mandelbrot–Li distribution is determined as

(29) $$p_{i}\left(L\right)=\frac{\gamma }{{\left(M+1\right)}^{L+2}}\;i=1,\ldots ,M^{L}$$

where γ is a normalization constant. Now, the summation of all probabilities of all such words is given by

(30) $$\begin{array}{c} \sum_{L=1}^{\infty }N_{w}\left(L\right)p_{i}\left(L\right)=1 \end{array}$$

(31) $$\begin{array}{c} =\sum_{L=1}^{\infty }\frac{\gamma M^{L}}{{\left(M+1\right)}^{L+2}} \end{array}$$

(32) $$\begin{array}{c} =\frac{\gamma M}{{\left(M+1\right)}^{2}} \end{array}$$

and hence the normalization constant can be found as

(33) $$\gamma =\frac{{\left(M+1\right)}^{2}}{M}$$

Li subsequently showed that, using an exponential transformation from the word length to word rank model, it is possible to derive a rank ordered, parametric probabilistic model-which extends the Zipf–Mandelbrot model and is defined in terms of the alphabet size *M* [30].

### 3.3. Constrained Linguistic Probabilistic Model

The model proposed by Li is particularly advantageous in a number of ways; however, in terms of our interest in synthetic language, the model makes a number of assumptions which depart from known statistical linguistics. For example, the model assumes that for a word of length *L*, the total number of words possible is $M^{L}$ given by (28). However, for a typical alphabet size, this vastly overestimates the number of words expected in a language, including many words which would not occur in known human languages.

As described in the previous section, the problem with the current ZML law is that the model is based on a simple estimate of the upper limit of possible words without consideration given to linguistic rules or other natural language principles beyond the initial power law. For example, in the English language, this might include orthographic spelling rules such as: (a) every word has at least one vowel; (b) “q” is almost always followed by “u”; (c) “s” never follows “x”; and (d) words never end in “v” or “j”. These could potentially be considered as priors in a model, and there are other aspects of interaction in human communication and natural languages beyond linguistics which could be considered as statistical principles (For convenience we refer to these broadly as linguistic constraints and note that they may be related to verbal or written language.)to include in a model.

Another view of this problem is in terms of optimal coding, where the aim is typically to encode the most frequently used words in the most efficient way [67,68]. Despite ongoing interest in this area, there is strong evidence for Zipf’s law of abbreviation which indicates that the highest ranking most frequent words tend to be shorter [38].

In contrast to a considerable body of work in deriving models to analyze and understand natural language, our interest is in deriving a probabilistic framework for constructing synthetic languages. Hence, in this section, we propose to consider a modified cZML law which is constrained to include linguistic principles.

As an example of linguistic features, we might consider the example of double-letter words. An extremely small number of words have double letters in comparison to the number of actual words possible. For five-letter English words, there are approximately 100 readily identifiable double letter words out of a possible $1.5\times 10^{18}$ words. Hence, we can introduce a new ZML model which introduces the constraint of not permitting words with adjacent double letters.

**Figure 3:**
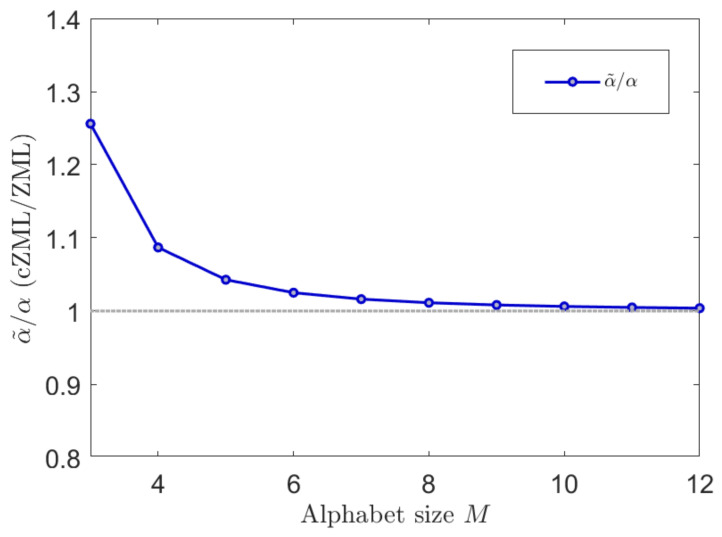
The effect of the proposed linguistic constraints on the modified ZML (cZML) vs. the usual ZML model are shown here by contrasting the relative parameters $\tilde{\alpha }/\alpha$ against M. It can be readily seen that the effect is most significant for smaller values of *M* but diminishes quickly as *M* increases, observed here for values up to M=12.

**Figure 4:**
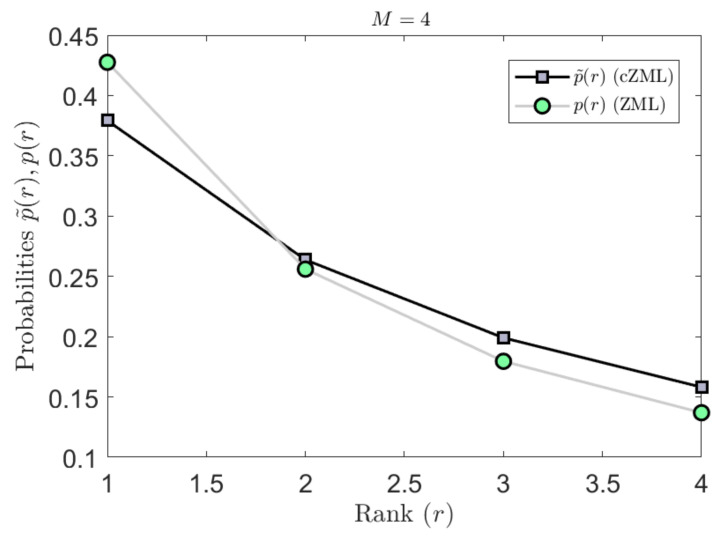
The effect of the proposed linguistic constraints on the modified cZML model are shown here by contrasting the ranked probabilities in each case. An example is shown here for M=4.

The derivation of the ZML with linguistic constraints is given in Appendix A.1. The effect of parametrization due to the constraints is indicated in Figure 3 where it can be readily seen that the effect is most significant for smaller values of *M* but diminishes quickly as *M* increases. Similarly, the effect of the constrained cZML model can be observed in Figure 4 where the change in the symbolic probabilities can be observed for small values of M.

This new cZML model introduces synthetic linguistic constraints based on having no adjacent repeating symbols. It is evident that other constraints can be considered to improve the accuracy of the model from a linguistics perspective, which we derive in the next section.

### 3.4. Constrained Linguistic Probabilistic Model II

This issue of the language space is well known in terms of language smoothing, where techniques such as the CN-gram (continuation n-gram [69]) have been proposed to reduce the space of possible words. Based on known human languages, the value of ${\tilde{N}}_{w}\left(L;M\right)$ used by the constrained ZML model considered above is generally too large for a given value of M.

Hence, in this section, we derived a constrained cZML which introduces a reduced lexicon space through a CN-gram-style approach. The derivation of the ZML with linguistic constraints in this case is given in Appendix A.2.

**Figure 5:**
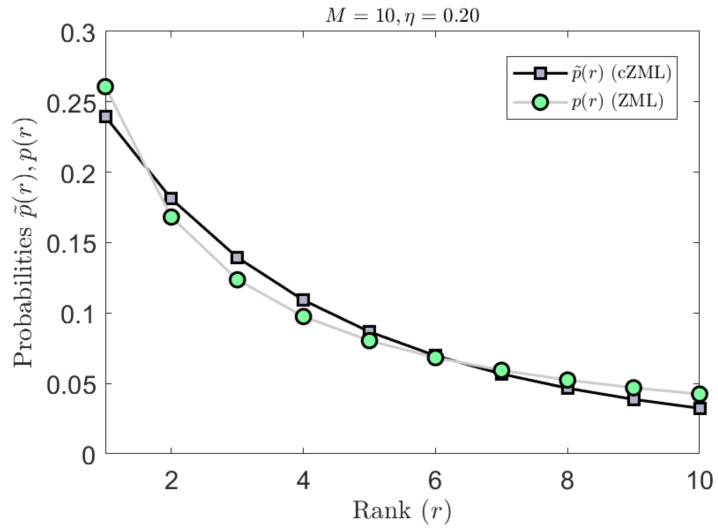
The second form of linguistically constrained cZML model has the effect of flattening the ranked probabilities. In the example shown here for M=10, it can be observed that the mid-ranked probabilities are increased, while the highest and lowest ranked probabilities are decreased.

The effect of the second form of the constrained ZML model can be observed in Figure 5 where the change in the symbolic probabilities can be observed for small values of M. While only a small probabilistic variation is observed, this corresponds to a significantly reduced maximum vocabulary size, which will lead to a more accurate estimation of entropy.

In the next section, we consider the performance of these newly proposed constrained ZML models within the efficient model-based entropy estimation algorithm described in Section 2.

## 4. Performance Results

### 4.1. Constrained Linguistic ZML Model for Natural Language

To test the performance of the proposed models, we applied them to English language data from the Google Web Trillion Word Corpus [70,71].

**Figure 6:**
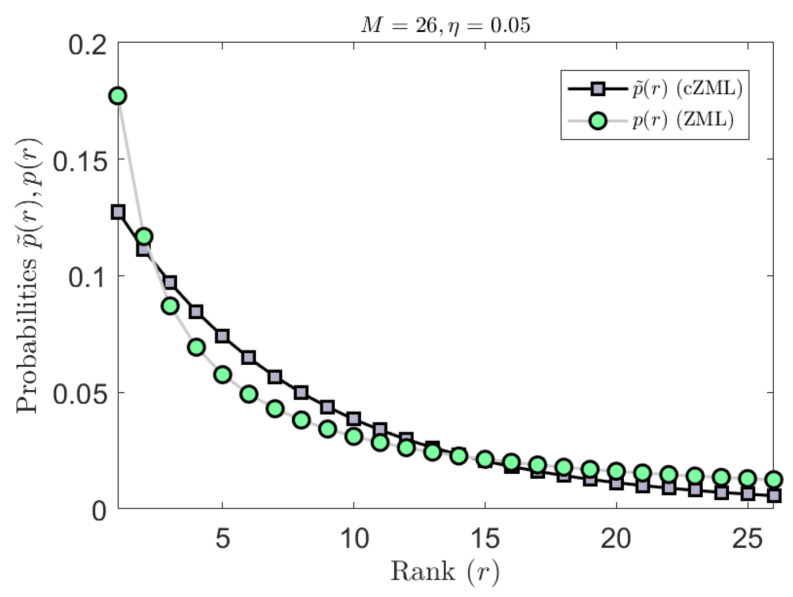
The second form of linguistically constrained cZML model has the effect of flattening the ranked probabilities. In the example shown here for M=26, it can be observed that the mid-ranked probabilities are increased, while the highest and lowest ranked probabilities are decreased.

**Figure 7:**
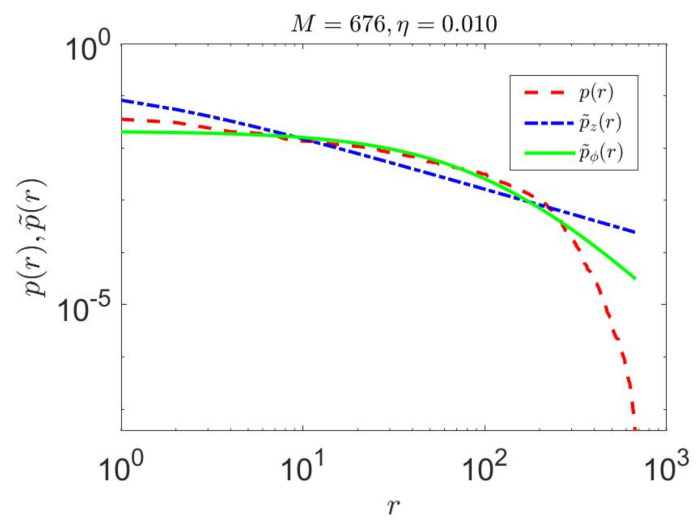
Performance of the constrained linguistic cZML model on actual English language data as compared to the unconstrained ZML model. In this case, we consider the full set of 676 two letter bigrams from the Google data set. The nonlinear behavior of the actual data is evidently (dashed curve) modeled with a higher degree of accuracy than the unconstrained model (dot dashed curve).

The proposed linguistically constrained cZML model approximates the higher ranked probabilities with better accuracy than the original ZML model and also enables better accuracy for the low-ranked probabilities (Figure 6 and Figure 7).

We consider the full set of 676 two letter bigrams from the Google data set. The nonlinear behavior of the actual data is evident (Figure 7). The original ZML model shows linear behavior and does not approximate the low ranked probabilities very well. In contrast, the proposed linguistically constrained cZML model has the effect of flattening the ranked probabilities to give better high-ranked approximation, while also producing more accurate behavior for the low ranked probabilities.

Note that since we utilize a model-based approach for estimating entropy, the choice of Zipfian model is significant to the outcome. Hence, because the proposed model more accurately approximates typical linguistic sequences, this can can be expected to lead to a more accurate entropy estimation process, though still within the limitations described above.

It is evident that, given the success of this approach, it is possible to consider numerous other constraints to improve the accuracy of the model from a linguistic perspective.

### 4.2. Entropy Rate Estimation

The entropy rate can be defined as the limit of joint entropy for an increasing number of symbols given by [67]

(34) $$\begin{array}{c} h_{r}\left(X\right)=\lim_{N\to \infty }\frac{1}{N}H_{N}\left(X\right) \end{array}$$

(35) $$\begin{array}{c} =\lim_{N\to \infty }\frac{1}{N}H_{N}\left(X_{1},\ldots ,X_{N}\right) \end{array}$$

where $X=X_{1},\ldots ,X_{N}$ can represent successive blocks of symbols. The task of entropy rate estimation is known to present a challenge due to the difficulty in obtaining a consistent estimate [72] and various number methods have been described. It is shown that, for the condition of stationarity, the entropy rate can be defined in terms of conditional entropy as [67]:

(36) $$h_{r}\left(X\right)=\lim_{N\to \infty }H_{N}\left(X_{N}|X_{1},\ldots ,X_{N-1}\right)$$

In practice, for finite sequences of symbols, the condition of stationarity may not hold and therefore estimating the entropy rate using (35) may result in a different value than with (36).

The value of entropy rate was estimated by Shannon using an experimental approach and found to be about one bit-per-character (bpc) [5]. Cover estimated the entropy rate for the novel *Jefferson the Virginian*, by Dumas Malone to be 1.25 bpc [73]. A word trigram method which used the cross-entropy between this model and a balanced sample of English text trained on a language model of 583 million symbols was applied to Form C of the Brown corpus which yielded an upper bound entropy rate estimate of 1.75 bpc [74]. A number of unigram entropy estimation methods using a stabilization criterion and a linear entropy to entropy rate conversion model were considered in terms of a large scale study across three parallel corpora, encompassing approximately 450M words in 1259 languages, leading to estimates of the entropy rate of 6 bits per word in [75]. An estimation method using the limit of successive backward differences in n-gram entropies was proposed in [76]. Compression algorithms have also been used as a basis for entropy rate estimation [77,78].

A method using an exponential extrapolation function was proposed in [79] to provide an estimate of entropy rate across multiple languages and 20 corpora provided results tending towards infinity. Interestingly, this result indicated that the entropy rates of human languages are positive but approximately 20% smaller than without extrapolation, which appears to be in agreement with the results obtained for entropy rate estimation using the algorithm proposed here.

Some issues are evident in the experimental studies because of the use of different entropy rate estimation algorithms, different corpora, the treatment of how n-grams are evaluated, for example whether only actually occurring n-grams within words are used or not (see for example the contrasting discussions between [3,74,75]), the inclusion of only alphabetic characters or whether to include punctuation, and various other factors.

Here, we estimate the entropy rate using the proposed cZML model-based entropy estimator applied to the Brown corpus which consists of approximately 5.5 million characters [80]. While a comprehensive comparison of the various entropy estimation algorithms is beyond the scope of this paper, the results of the proposed algorithm are compared with the plug-in entropy estimation approach. Prefiltering was performed to remove all non-alphanumeric characters except for spaces, and n-grams which are not part of any word were excluded. It is well recognized that a smaller data set presents a challenge for entropy estimation especially with increasing word lengths [3,75] and so it is of interest to observe the relative performance of the proposed algorithm.

The conditional entropy rate estimation approach of (36) was adopted in each case. For the entire Brown corpus, the entropy rate was estimated as 1.29 bpc, whereas using plug-in method, the result was 2.04 bpc and outside the upper bound indicated in [74]. The results from the proposed algorithm compare well with the result of 1.25 bpc for a large collection of English corpora in [3]; however, it is evident that the results from the plug-in method are significantly different, giving less confidence in their reliability. The advantage of requiring fewer samples for the proposed entropy estimation algorithm is also apparent in this case of entropy rate estimation.

### 4.3. Convergence of Constrained cZML Entropy Estimation Algorithm

The convergence characteristics of the proposed algorithm can be thereby obtained by generating a random sequence of symbols according to $\left\{p_{j}\left(x\right)\right\}$ and the estimated entropy is developed as a function of the sample size $N_{s}$.

**Figure 8:**
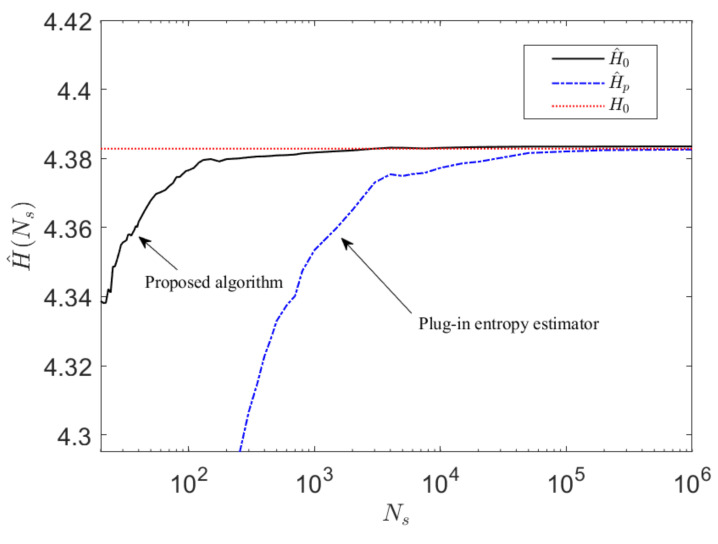
The convergence performance of the proposed entropy estimation algorithm is compared to a regular plug-in estimator for M=30 as a function of the sample size, up to $N_{s}=10^{6}$ samples averaged across $N_{v}=75$ trials. It can be noted that the estimate ${\hat{H}}_{0}\left(N_{s}\right)$ from the proposed algorithm converges rapidly very closely towards the true entropy value (within three decimal places). In contrast, the conventional plug-in estimator ${\hat{H}}_{p}\left(N_{s}\right)$ converges much more slowly towards a biased estimate.

This approach is applied to an example case where M=30 with the results shown in Figure 8. The performance of the proposed algorithm is compared with a conventional plug-in estimator on a defined entropy estimation task over a large range of samples sizes up to $N_{s}=10^{6}$ symbolic samples. The mean entropy estimate is obtained by averaging over $N_{v}=75$ trials, where it can be observed that the new algorithm converges very rapidly, requiring significantly fewer samples to converge compared to a conventional plug-in estimator approach [14].

Note that since both the proposed algorithm and the conventional plug-in entropy estimation algorithm rely on a maximum likelihood method, the computational burden of each is O(N). However, the key advantage of the proposed model-based algorithm is that it requires substantially fewer samples than the conventional plug-in entropy estimator.

It is well known that conventional plug-in estimators will give biased estimates. For the proposed algorithm, any such bias will result from the accuracy with which $D_{r}\left(M\right)$ can be estimated. It can be noted that as with conventional probability estimates, provided the system is stationary, it is possible to improve the estimate of $D_{r}\left(M\right)$ by increasing the number of samples.

## 5. Conclusions

Entropy estimators which go beyond naive maximum likelihood methods are of considerable interest, particularly in terms of overcoming limitations due to data. The approach of coincidence counting is recognized as a potentially powerful approach for model-based estimators. Here, we show that an efficient coincidence counting estimator can be derived using a Zipf–Mandelbrot–Li law which provides a significant reduction in the data required.

Interestingly, while Zipfian laws are ubiquitous in fields such as natural language, surprisingly, it appears that these models have evidently been developed essentially without particular regard for the possible inclusion of linguistic constraints.

In this paper, by introducing some simple linguistic constraints, we extended the regular rank-based Zipf–Mandelbrot–Li model to one which provides more realistic assumptions and makes it more suitable for a broad range of probabilistic language models which rely on an analytical Zipfian framework. Such models can be applied to the human language and provide a necessary foundation for developing synthetic language models.

Conceptually, the idea of model-based entropy estimators seems like it may potentially sacrifice accuracy; however, our results show that for natural systems where the symbolic events follow an approximate Zipfian distribution and using limited data, the performance is better than that obtained by a naive entropy estimator. We derived results which indicate that the expected improvement in convergence and demonstrated the efficacy of the proposed model on the entropy rate estimation and two-letter bigram entropy estimation where it was shown to produce more accurate behavior for both low-ranked and high-ranked symbolic probabilities.

The proposed constrained linguistic Zipf–Mandelbrot–Li model appears to be the first time this approach has been adopted. In future work, it would be of interest to further extend this concept by introducing more sophisticated linguistic constraints and to explore applications where limited data are available.

## Data Availability

Data used for Section 4.1, derived from the Google Web Trillon Word Corpus is available from https://norvig.com/ngrams (accessed on 18 August 2021). The Brown corpus used in Section 4.2 is available as part of the NLTK from https://www.nltk.org/book/ch02.html (accessed on 18 August 2021).

## Appendix A. Derivation of Constrained Linguistic Zipf–Mandelbrot–Li Models

### Appendix A.1. cZML Model I

The Zipf–Mandelbrot–Li law with linguistic constraints of the first type is derived as follows. For a word of length *L*, which is constrained to have no repeating adjacent symbols, the total number of words possible is given by

(A1) $$\begin{array}{cc} {\tilde{N}}_{w} & =M\prod_{k=1M}^{L}\left(M_{k}-1\right),\;k=2,\ldots ,L\\ \; & =M{\left(M-1\right)}^{L-1} \end{array}$$

and the frequency of occurrence for a word of length *L* using the constrained probabilistic model is:

(A2) $${\tilde{p}}_{i}\left(L\right)=\frac{\tilde{\gamma }}{\left(M+1\right)M^{L+1}}\;i=1,\ldots ,{\tilde{N}}_{w}^{}\left(L\right)$$

where $\tilde{\gamma }$ is a normalization constant. It follows that $\tilde{\gamma }$ can be determined from the word frequency normalization condition via the summation of all probabilities of such words [30], hence:

(A3) $$\begin{array}{c} \sum_{L=1}^{\infty }{\tilde{N}}_{w}\left(L\right){\tilde{p}}_{i}\left(L\right)=1 \end{array}$$

(A4) $$\begin{array}{c} =\sum_{L=1}^{\infty }\frac{\tilde{\gamma }{\left(M-1\right)}^{L-1}}{\left(M+1\right)M^{L}} \end{array}$$

(A5) $$\begin{array}{c} =\frac{\tilde{\gamma }}{\left(M+1\right)} \end{array}$$

and hence the normalization constant can be found as

(A6) $$\tilde{\gamma }=M+1$$

From (A2), the probability for any possible constrained word is:

(A7) $${\tilde{p}}_{i}\left(L\right)=\frac{1}{M^{L+1}}\;i=1,\ldots ,{\tilde{N}}_{w}\left(L\right)$$

and hence the frequency of occurrence of all words of length *L* and with the constraint that there are no adjacent repeating symbols is given by an exponential function of *L* as

(A8) $$\begin{array}{cc} \tilde{p}\left(L\right) & =M{\left(M-1\right)}^{L-1}{\tilde{p}}_{i}\left(L\right) \end{array}$$

(A9) $$\begin{array}{cc} \; & =\frac{{\left(M-1\right)}^{L-1}}{M^{L}} \end{array}$$

Now it follows that the rank can be considered either in terms of the direct probability or equivalently, as the incremental change in probability as we change the word length, i.e., from L−1 to L. Consider the rank of probabilities in the exponentially decreasing distribution $\left\{p_{i}\left(M,L\right)\right\},$ defined as

(A10) $$r\left(i;\left\{p_{i}\left(M,L\right)\right\}\right)=\arg_{i}\left(\left\{p_{i}\left(M,L\right)\right\}\right)$$

then from (A2) it follows that:

(A11) $$p_{i}\left(L\right)<p_{i}\left(L+1\right)$$

and hence:

(A12) r(L)>r(L+1)

where it is evident that the rank r(L) is proportional to an inverse function *g* of the corresponding probabilities, such that:

(A13) $$\begin{array}{cc} r(L) & =g\left(M,L\right)\\ \; & \leq M^{L} \end{array}$$

Hence, since the rank is effectively determined by the inverse of the probabilities, and the probabilities are found as the inverse of the number of occurrences, and accordingly, we can calculate the total number of cumulative occurrences for all words up to length L, which in turn provides a measure of the ranking. Therefore, we have:

(A14) $$\sum_{k=1w}^{L-1}{\tilde{N}}_{w}\left(k\right)<r\left(L\right)\leq \sum_{k=1}^{L}{\tilde{N}}_{w}\left(k\right)$$

where:

(A15) $$\begin{array}{cc} \sum_{k=1}^{L-1}{\tilde{N}}_{w}\left(k\right) & =\sum_{k=1}^{L-1}M{\left(M-1\right)}^{k-1} \end{array}$$

(A16) $$\begin{array}{cc} \; & =\frac{M\left({\left(M-1\right)}^{L-1}-1\right)}{M-2} \end{array}$$

and:

(A17) $$\begin{array}{cc} \sum_{k=1}^{L}{\tilde{N}}_{w}\left(k\right) & =\sum_{k=1}^{L}M{\left(M-1\right)}^{k-1} \end{array}$$

(A18) $$\begin{array}{cc} \; & =\frac{M\left({\left(M-1\right)}^{L}-1\right)}{M-2} \end{array}$$

and hence:

(A19) $$\frac{M\left({\left(M-1\right)}^{L-1}-1\right)}{M-2}<r\left(L\right)\leq \frac{M\left({\left(M-1\right)}^{L}-1\right)}{M-2}$$

which represents the exponential transformation from the constrained word’s length to the word’s rank under the given constraint:

Rearranging (A19) and taking logs, for the lower bound, we have:

(A20) $$\begin{array}{c} \frac{\left(M-2)r(L\right)}{M}>{\left(M-1\right)}^{L-1}-1 \end{array}$$

(A21) $$\begin{array}{c} L-1<\log_{M-1}\left(\frac{\left(M-2)r(L\right)}{M}+1\right) \end{array}$$

and for the upper bound, we have:

(A22) $$\begin{array}{c} \frac{\left(M-2)r(L\right)}{M}\leq {\left(M-1\right)}^{L}-1 \end{array}$$

(A23) $$\begin{array}{c} L\geq \log_{M-1}\left(\frac{\left(M-2)r(L\right)}{M}+1\right) \end{array}$$

Following a similar approach to [30], raising $\frac{1}{M}$ to the power of the terms in (A21)–(A23) gives:

(A24) $$\frac{1}{M^{L-1}}>{\left(\frac{1}{M}\right)}^{\varsigma }\geq \left(\frac{1}{M^{L}}\right)$$

where:

(A25) $$\varsigma =\log_{M-1}\left(\frac{\left(M-2)r(L\right)}{M}+1\right)$$

Using the identity

(A26) $${\left(\frac{1}{a}\right)}^{\log b}={\left(\frac{1}{b}\right)}^{\log a}$$

then, it follows using a change of base:

(A27) $$\begin{array}{c} {\left(\frac{1}{M}\right)}^{\varsigma }={\left(\frac{1}{\left(\frac{M-2}{M}\right)r\left(L\right)+1}\right)}^{\log_{M-1}\left(M\right)} \end{array}$$

(A28) $$\begin{array}{c} ={\left(\frac{1}{\left(\frac{M-2}{M}\right)r\left(L\right)+1}\right)}^{\frac{\log\left(M\right)}{\log\left(M-1\right)}} \end{array}$$

noting that:

(A29) $$\frac{1}{MM^{L-1}}=\frac{1}{M^{L}}={\tilde{p}}_{i}\left(L-1\right)$$

(A30) $$\frac{1}{MM^{L}}=\frac{1}{M^{L+1}}={\tilde{p}}_{i}\left(L\right)$$

then (A24) can be multiplied by 1/M to give probabilistic bounds, and hence we have:

(A31) $$\begin{array}{c} \frac{1}{M}{\left(\frac{1}{\left(\frac{M-2}{M}\right)r\left(L\right)+1}\right)}^{\tilde{\alpha }}=\frac{\frac{1}{M}{\left(\frac{M}{M-2}\right)}^{\tilde{\alpha }}}{{\left(r\left(L\right)+\frac{M}{M-2}\right)}^{\tilde{\alpha }}} \end{array}$$

(A32) $$\begin{array}{c} =\frac{\frac{M^{\tilde{\alpha }-1}}{{\left(M-2\right)}^{\tilde{\alpha }}}}{{\left(r\left(L\right)+\frac{M}{M-2}\right)}^{\tilde{\alpha }}} \end{array}$$

(A33) $$\begin{array}{c} =\frac{\tilde{\gamma }}{{\left(r\left(L\right)+\tilde{\beta }\right)}^{\tilde{\alpha }}} \end{array}$$

where:

(A34) $$\begin{array}{cc} \tilde{\alpha } & =\frac{\log\left(M\right)}{\log\left(M-1\right)} \end{array}$$

(A35) $$\begin{array}{cc} \tilde{\beta } & =\frac{M}{M-2} \end{array}$$

(A36) $$\begin{array}{cc} \tilde{\gamma } & =\frac{M^{\tilde{\alpha }-1}}{{\left(M-2\right)}^{\tilde{\alpha }}} \end{array}$$

which leads to:

(A37) $${\tilde{p}}_{i}\left(L-1\right)<\frac{\tilde{\gamma }}{{\left(r\left(L\right)+\tilde{\beta }\right)}^{\tilde{\alpha }}}\leq {\tilde{p}}_{i}\left(L\right)$$

which can be considered as a new form of Zipf–Mandelbrot–Li law which includes the specified linguistic constraints such that the constants originally computed according to (12)–(16), are now computed according to (A34)–(A36).

Note that as the alphabet size *M* increases, the parameter given by (A34) converges to that of (12) in the original formulation:

(A38) $$\lim_{M\to \infty }\frac{\tilde{\alpha }}{\alpha }=1$$

A formulation of the model using a maximum word length $L_{\max}$ can now be introduced. It follows that a new value for $\tilde{\gamma }$ can be determined from the word frequency normalization condition via the summation of all probabilities as before, hence:

(A39) $$\begin{array}{c} \sum_{L=1}^{L_{\max}}{\tilde{N}}_{w}\left(L\right){\tilde{p}}_{i}\left(L\right)=1 \end{array}$$

(A40) $$\begin{array}{c} =\sum_{L=1}^{L_{\max}}\frac{\tilde{\gamma }{\left(M-1\right)}^{L-1}}{\left(M+1\right)M^{L}} \end{array}$$

which can be shown to reduce to:

(A41) $$\begin{array}{c} \frac{\tilde{\gamma }}{M+1}-\frac{\tilde{\gamma }M{\left(\frac{M-1}{M}\right)}^{\left(L_{\max}+1\right)}}{\left(M+1\right)\left(M-1\right)}=1 \end{array}$$

(A42) $$\begin{array}{c} =\frac{\tilde{\gamma }\left(1-{\left(\frac{M-1}{M}\right)}^{L\max}\right)}{M+1} \end{array}$$

and hence:

(A43) $$\tilde{\gamma }=\frac{M+1}{1-{\left(\frac{M-1}{M}\right)}^{L_{\max}}}$$

### Appendix A.2. cZML Model II

The Zipf–Mandelbrot–Li law with linguistic constraints of the second type is derived as follows. For a word of length *L*, the total number of words possible is given by

(A44) $$\begin{array}{cc} {\tilde{N}}_{w} & =M\prod_{k=1}^{L-1}\eta \left(M_{k}-1\right),\;k=1,\ldots ,L\\ \; & =\eta^{L-1}M{\left(M-1\right)}^{L-1} \end{array}$$

and hence the frequency of occurrence for a word of length *L* using the constrained probabilistic model is

(A45) $${\tilde{p}}_{i}\left(L\right)=\frac{\tilde{\gamma }}{\left(M+1\right){\left(\eta M\right)}^{L+1}}\;i=1,\ldots ,{\tilde{N}}_{w}^{}\left(L\right)$$

where: $\tilde{\gamma }$ is a normalization constant. It follows that $\tilde{\gamma }$ can be determined from the word frequency normalization condition via the summation of all probabilities of such words [30], hence:

(A46) $$\begin{array}{c} \sum_{L=1}^{\infty }{\tilde{N}}_{w}\left(L\right){\tilde{p}}_{i}\left(L\right)=1 \end{array}$$

(A47) $$\begin{array}{c} =\sum_{L=1}^{\infty }\frac{\tilde{\gamma }\eta^{L-1}M{\left(M-1\right)}^{L-1}}{\left(M+1\right){\left(\eta M\right)}^{L+1}} \end{array}$$

(A48) $$\begin{array}{c} =\sum_{L=1}^{\infty }\frac{\tilde{\gamma }\eta^{-2}M^{-L}{\left(M-1\right)}^{L-1}}{\left(M+1\right)} \end{array}$$

(A49) $$\begin{array}{c} =\frac{\tilde{\gamma }}{\eta^{2}\left(M+1\right)} \end{array}$$

and hence the normalization constant can be found as

(A50) $$\tilde{\gamma }=\eta^{2}\left(M+1\right)$$

From (A45), the probability for any possible constrained word is

(A51) $${\tilde{p}}_{i}\left(L\right)=\frac{1}{\eta^{L-1}M^{L+1}}\;\;i=1,\ldots ,{\tilde{N}}_{w}\left(L\right)$$

and hence the frequency of the occurrence of all words of length *L* and with the constraint that there are no adjacent repeating symbols is given by an exponential function of *L* as

(A52) $$\begin{array}{cc} \tilde{p}\left(L\right) & =\eta^{L-1}M{\left(M-1\right)}^{L-1}{\tilde{p}}_{i}\left(L\right) \end{array}$$

(A53) $$\begin{array}{cc} \; & =\frac{{\left(M-1\right)}^{L-1}}{M^{L}} \end{array}$$

Now it follows that the rank can be considered either in terms of direct probability or equivalently, as the incremental change in probability as we change the word length, i.e., from L−1 to L. Consider the rank of probabilities in the exponentially decreasing distribution $\left\{p_{i}\left(M,L\right)\right\},$ defined as

(A54) $$r\left(i;\left\{p_{i}\left(M,L\right)\right\}\right)=\arg_{i}\left(\left\{p_{i}\left(M,L\right)\right\}\right)$$

then, from (A45), it follows that:

(A55) $$p_{i}\left(L\right)<p_{i}\left(L+1\right)$$

and hence:

(A56) r(L)>r(L+1)

where it is evident that the rank r(L) is proportional to an inverse function *g* of the corresponding probabilities such that:

(A57) $$\begin{array}{cc} r(L) & =g\left(M,L\right)\\ \; & \leq M^{L} \end{array}$$

Hence, since the rank is effectively determined by the inverse of the probabilities, and the probabilities are found as the inverse of the number of occurrences, accordingly, we can calculate the total number of cumulative occurrences for all words up to length L, which in turn provides a measure of the ranking. Therefore, we have:

(A58) $$\sum_{k=1w}^{L-1}{\tilde{N}}_{w}\left(k\right)<r\left(L\right)\leq \sum_{k=1}^{L}{\tilde{N}}_{w}\left(k\right)$$

where:

(A59) $$\begin{array}{cc} \sum_{k=1}^{L-1}{\tilde{N}}_{w}\left(k\right) & =\sum_{k=1}^{L-1}\eta^{k-1}M{\left(M-1\right)}^{k-1} \end{array}$$

(A60) $$\begin{array}{cc} \; & =\frac{M\left(\eta^{L}{\left(M-1\right)}^{L}-\eta \left(M-1\right)\right)}{\eta \left(M-1\right)\left(\eta \left(M-1\right)-1\right)} \end{array}$$

(A61) $$\begin{array}{cc} \; & =\frac{M\left(\eta^{L-1}{\left(M-1\right)}^{L-1}-1\right)}{\eta \left(M-1\right)-1} \end{array}$$

and:

(A62) $$\begin{array}{cc} \sum_{k=1}^{L}{\tilde{N}}_{w}\left(k\right) & =\sum_{k=1}^{L}\eta^{k-1}M{\left(M-1\right)}^{k-1} \end{array}$$

(A63) $$\begin{array}{cc} \; & =\frac{M(\eta^{L}{\left(M-1\right)}^{L}-1)}{\left.\eta \left(M-1\right)-1\right.} \end{array}$$

and hence:

(A64) $$\frac{M\left(\eta^{L-1}{\left(M-1\right)}^{L-1}-1\right)}{\eta \left(M-1\right)-1}<r\left(L\right)\leq \frac{M(\eta^{L}{\left(M-1\right)}^{L}-1)}{\eta \left(M-1\right)-1}$$

which represents the exponential transformation from the constrained word’s length to the word’s rank under the given constraint. Rearranging (A64) and taking logs, we have:

(A65) $$\begin{array}{c} \frac{\left(\eta \left(M-1\right)-1\right)r\left(L\right)}{M}>\eta^{L-1}{\left(M-1\right)}^{L-1}-1 \end{array}$$

(A66) $$\begin{array}{c} L-1<\log_{\eta \left(M-1\right)}\left(\frac{\left(\eta \left(M-1\right)-1\right)r\left(L\right)}{M}+1\right) \end{array}$$

and for the upper bound, we have:

(A67) $$\begin{array}{c} \frac{\left(\eta \left(M-1\right)-1\right)r\left(L\right)}{M}\leq {\left(M-1\right)}^{L}-1 \end{array}$$

(A68) $$\begin{array}{c} L\geq \log_{\eta \left(M-1\right)}\left(\frac{\left(\eta \left(M-1\right)-1\right)r\left(L\right)}{M}+1\right) \end{array}$$

Following a similar approach to [30], raising $\frac{1}{M}$ to the power of the bound terms in (A65)–(A68) gives:

(A69) $$\frac{1}{M^{L-1}}>{\left(\frac{1}{M}\right)}^{\varsigma }\geq \left(\left(\frac{1}{M^{L}}\right)\right)$$

where:

(A70) $$\varsigma =\log_{\eta \left(M-1\right)}\left(\frac{\left(\eta \left(M-1\right)-1\right)r\left(L\right)}{M}+1\right)$$

Using the identity:

(A71) $${\left(\frac{1}{a}\right)}^{\log b}={\left(\frac{1}{b}\right)}^{\log a}$$

then it follows using a change of base:

(A72) $$\begin{array}{c} {\left(\frac{1}{M}\right)}^{\varsigma }={\left(\frac{1}{\left(\frac{\eta \left(M-1\right)-1}{M}\right)r\left(L\right)+1}\right)}^{\log_{\eta \left(M-1\right)}\left(M\right)} \end{array}$$

(A73) $$\begin{array}{c} ={\left(\frac{1}{\left(\frac{\eta \left(M-1\right)-1}{M}\right)r\left(L\right)+1}\right)}^{\frac{\log\left(M\right)}{\log\eta \left(M-1\right)}} \end{array}$$

Noting that:

(A74) $$\frac{1}{MM^{L-1}}=\frac{1}{M^{L}}={\tilde{p}}_{i}\left(L-1\right)$$

(A75) $$\frac{1}{MM^{L}}=\frac{1}{M^{L+1}}={\tilde{p}}_{i}\left(L\right)$$

then (A69) can be multiplied by 1/M to give probabilistic bounds, and hence we have:

(A76) $$\begin{array}{c} \frac{1}{M}{\left(\frac{1}{\left(\frac{\eta \left(M-1\right)-1}{M}\right)r\left(L\right)+1}\right)}^{\tilde{\alpha }}=\frac{\frac{1}{M}{\left(\frac{M}{\eta \left(M-1\right)-1}\right)}^{\tilde{\alpha }}}{{\left(r\left(L\right)+\frac{M}{\eta \left(M-1\right)-1}\right)}^{\tilde{\alpha }}} \end{array}$$

(A77) $$\begin{array}{c} =\frac{\frac{M^{\tilde{\alpha }-1}}{{\left(\eta \left(M-1\right)-1\right)}^{\tilde{\alpha }}}}{{\left(r\left(L\right)+\frac{M}{\eta \left(M-1\right)-1}\right)}^{\tilde{\alpha }}} \end{array}$$

(A78) $$\begin{array}{c} =\frac{\tilde{\gamma }}{{\left(r\left(L\right)+\tilde{\beta }\right)}^{\tilde{\alpha }}} \end{array}$$

where:

(A79) $$\begin{array}{cc} \tilde{\alpha } & =\frac{\log\left(M\right)}{\log\eta \left(M-1\right)} \end{array}$$

(A80) $$\begin{array}{cc} \tilde{\beta } & =\frac{M}{\eta \left(M-1\right)-1} \end{array}$$

(A81) $$\begin{array}{cc} \tilde{\gamma } & =\frac{M^{\tilde{\alpha }-1}}{{\left(\eta \left(M-1\right)-1\right)}^{\tilde{\alpha }}} \end{array}$$

which leads to:

(A82) $${\tilde{p}}_{i}\left(L-1\right)<\frac{\tilde{\gamma }}{{\left(r\left(L\right)+\tilde{\beta }\right)}^{\tilde{\alpha }}}\leq {\tilde{p}}_{i}\left(L\right)$$

which can be considered as a further linguistically constrained form of the Zipf–Mandelbrot–Li law, which includes the specified linguistic constraints such that the constants originally computed according to (12)–(16) are now computed according to (A79)–(A81).

Note that as before, as the alphabet size *M* increases, the parameter given by (A79) converges to that of (12) in the original formulation:

(A83) $$\lim_{M\to \infty }\frac{\tilde{\alpha }}{\alpha }=1$$

and the effect of the parametrization due to the constraints is similar to the first case.

Following the same approach as before, a formulation of the model using a maximum word length $L_{\max}$ can now be introduced. It follows that a new value for $\tilde{\gamma }$ can be determined from the word frequency normalization condition via the summation of all probabilities as before, hence:

$$\begin{array}{c} \sum_{L=1}^{L_{\max}}{\tilde{N}}_{w}\left(L\right){\tilde{p}}_{i}\left(L\right)=1 \end{array}$$

(A84) $$\begin{array}{c} =\sum_{L=1}^{L_{\max}}\frac{\tilde{\gamma }{\left(M-1\right)}^{L-1}}{\left(M+1\right)M^{L}} \end{array}$$

(A85) $$\begin{array}{c} =\sum_{L=1}^{L_{\max}}\frac{\tilde{\gamma }\eta^{-2}M^{-L}{\left(M-1\right)}^{L-1}}{\left(M+1\right)} \end{array}$$

which can be shown to reduce to:

(A86) $$\begin{array}{c} \frac{\tilde{\gamma }}{M+1}-\frac{\tilde{\gamma }M{\left(\frac{M-1}{M}\right)}^{\left(L_{\max}+1\right)}}{\left(M+1\right)\left(M-1\right)}=1 \end{array}$$

(A87) $$\begin{array}{c} \frac{\tilde{\gamma }\left(1-{\left(\frac{M-1}{M}\right)}^{L\max}\right)}{\eta^{2}\left(M+1\right)}=1 \end{array}$$

and hence:

(A88) $$\tilde{\gamma }=\frac{\eta^{2}\left(M+1\right)}{1-{\left(\frac{M-1}{M}\right)}^{L_{\max}}}$$

This ZML model introduces further synthetic linguistic constraints based on having no adjacent repeating symbols.
